# Iron Deficiency – Not Only a Premenopausal Topic After Bariatric Surgery?

**DOI:** 10.1007/s11695-021-05380-3

**Published:** 2021-04-05

**Authors:** Ines Kunst, Michael Krebs, Bettina Dreschl, Gerhard Prager, Elias Meyer, Alexandra Kautzky-Willer, Tamara Ranzenberger-Haider

**Affiliations:** 1 Division of Endocrinology and Metabolism, Department of Internal Medicine III, Medical University of Vienna, Währinger Gürtel 18-20, 1090 Vienna, Austria; 2 Division of Gastroenterology and Hepatology, Department of Internal Medicine III, Nutritional Counselling, Medical University of Vienna, Währinger Gürtel 18-20, 1090 Vienna, Austria; 3 Division of Visceral Surgery, Department of General Surgery, Medical University of Vienna, Währinger Gürtel 18-20, 1090 Vienna, Austria; 4 Center for Medical Statistics, Informatics and Intelligent Systems (CeMSIIS), Medical University of Vienna, Spitalgasse 23, BT88, 1090 Vienna, Austria

**Keywords:** Bariatric surgery, Follow-up, Iron supplementation, Iron deficiency, Menopause

## Abstract

**Purpose:**

In our centre, specialized high dose multivitamin supplementation designed to meet the needs of patients after gastric bypass surgery is routinely recommended in the early postoperative period. The aim of the present study was to analyse whether iron supplementation prescribed in clinical practice is sufficient in both sexes and whether multivitamin supplementation standardized for women might potentially lead to iron overload in men.

**Materials/Methods:**

This was a retrospective study covering the period up to 36 months after bariatric surgery. Three groups were compared (men, premenopausal and postmenopausal women). The iron status was evaluated employing serum ferritin concentrations.

**Results:**

A total of 283 patients who had at least one follow-up visit between January 2015 and April 2018 at a specialized academic outpatient centre were included (71 men, 130 premenopausal women, 82 postmenopausal women). Thirty-six months after surgery, 33.3%, 68.4% and 54.5% of the men, pre- and postmenopausal women, respectively, were iron deficient. The preoperative prevalence of excess ferritin levels was 13.7% in premenopausal, 3.0% in postmenopausal women, 5.7% in men and declined in the following months.

**Conclusion:**

Iron deficiency is very common after gastric bypass surgery, and even high dosages of multivitamin and mineral supplements might not be sufficient to prevent the development of iron deficiency. Men, pre- and postmenopausal women differ in their prevalence of iron deficiency which demands adapted iron dosage regimens based on the sex and the age. Iron overload is rare in all observed groups and highest in premenopausal women.

**Graphical abstract:**

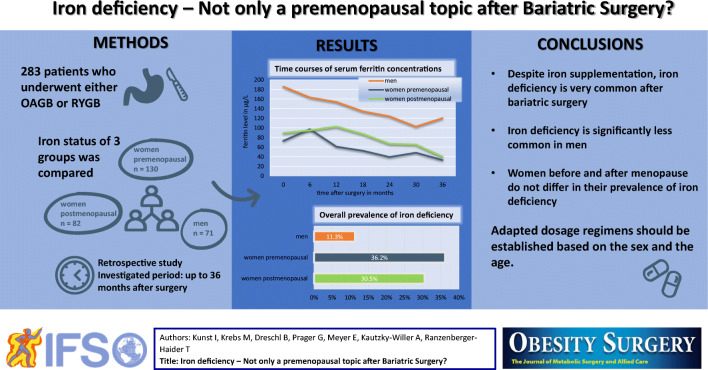

## Introduction

Bypassing large parts of the intestine warrants a lower energy absorption and together with changes in the secretion of gastrointestinal hormones provides for losing weight after bariatric surgery [1]. Apart from the benefits that go beyond losing weight and lead to major improvements in conditions associated with obesity [2], the changes in the digestive tract are also the reason for the occurrence of deficiencies in macro- and micronutrients after surgery [3]. Especially the patient collective seeking bariatric surgery already suffers from deficiencies prior to surgery, because despite excessive dietary consumption, the diet of people with obesity is often energy dense but at the same time poor in its nutritional value [4]. Iron deficiency is the most frequent mineral deficiency worldwide [5] and also very common after bariatric surgery. The small remaining stomach produces lesser gastric acid, and parts of the duodenum and upper jejunum are bypassed which both result in poorer iron absorption [3, 6, 7].

We sought to evaluate the iron status of the patients in our department after a bariatric procedure regarding the intake of iron supplements. A special focus of this study lies on sex- and menopause-related differences and whether there is a possible requirement of implementing a sex-specific substitution regimen. Most studies focus merely on deficiencies, but the development of excessive levels is also of a certain possibility due to over-supplementation.

## Methods

**Fig. 1:**
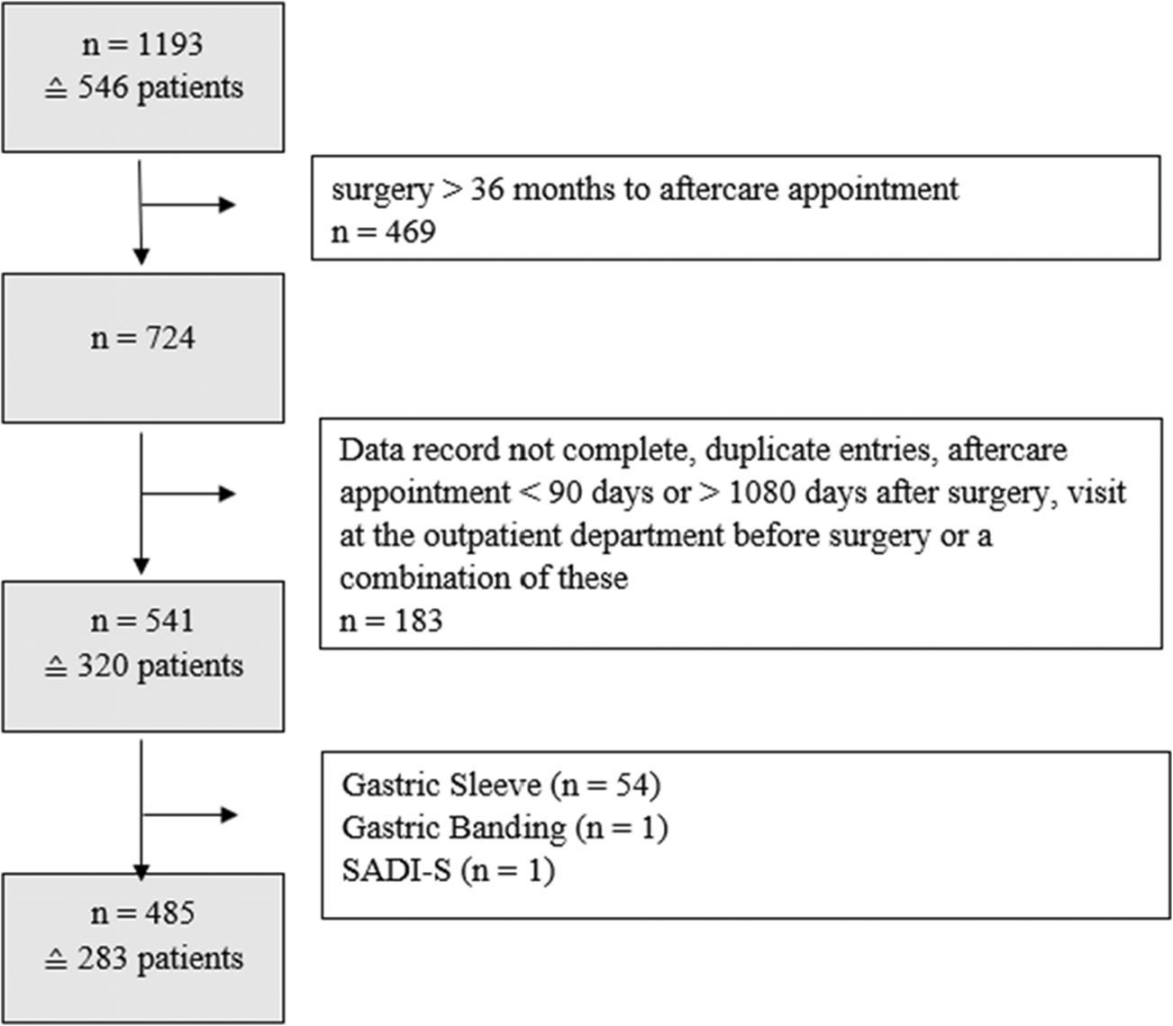
Database flowchart

A retrospective exploratory data analysis of a database of patients who underwent bariatric surgery with a gastric bypass procedure was performed. Inclusion criteria were a history of either one anastomosis gastric bypass (OAGB) or Roux-en-Y gastric bypass (RYGB) and a minimum of one follow-up examination 3 to 36 months after surgery with documented ferritin values between January 2015 and April 2018 at the Department of Medicine III in the Division of Endocrinology and Metabolism (Medical University of Vienna) (Fig. 1). The bypassed small bowel length (biliopancreatic limb) was 150 cm in all patients (RYGB and OAGB). The study protocol was approved by the ethics committee of the Medical University of Vienna (1361/2018).

All pre- and postoperative data was collected in an Obesity Register of the Medical University of Vienna. These data included the date of the follow-up visits, anthropometric measurements, laboratory investigations, type of surgery, prescription of vitamin and mineral supplementation and dosage, and other medication.

The following time periods were analysed in this present study: preoperative, 6 (± 3), 12 (± 3), 18 (± 3), 24 (± 3), 30 (± 3) and 36 (−3) months after surgery. If a patient visited more than once in one of these time periods, the data of the last available visit was used for the analysis. The patients were divided into three groups (men, pre- and postmenopausal women); the menopausal status was assessed by measurements of the luteinizing hormone level and the follicle-stimulating hormone level at every visit. The reference range of the General Hospital Vienna (www.kimcl.at) was used, and menopause was defined as a luteinizing hormone level above 7.7 mIU/mL and a follicle-stimulating hormone above 25.8 mIU/mL.

Prescription of daily oral multivitamin and mineral supplements was standardized only at discharge after surgery, where supplements specialized for patients after bariatric surgeries containing up to 70 mg of elemental iron were recommended on a routine basis. In the following aftercare, prescriptions and intake varied depending on patients’ preferences and prescriptions by diverse physicians including family doctors and our outpatient department.

All laboratory parameters were assessed by using routine laboratory methods (www.kimcl.at). The values above or below the reference range of the hospital laboratory of the General Hospital Vienna were used as the definition of a nutritional excess or deficiency level. To evaluate the iron status, the serum ferritin level with the following reference ranges was used: premenopausal women 15–150 μg/L and postmenopausal women and men 30–400 μg/L.

The changes in weight in this study are expressed as body mass index (BMI, a person’s bodyweight in relation to their height (kg/m^2^)) and the relative total weight loss (%TWL, the weight loss in relation to the total weight before surgery). The authors propose that ≥ 20% TWL can be considered a good result after bariatric surgery [8].

Exploratory statistical analysis was performed using SPSS (IBM, Version 26) and Microsoft Excel (Microsoft 365). The longitudinal data is presented as mean ± standard deviation (SD) or absolute and relative numbers at each investigated period. Due to perceived non-normality of the data, non-parametric tests were applied to compare the preoperative characteristics and weight parameter changes of the three groups (Kruskal-Wallis-Test or Wilcoxon signed rank test). Differences between the groups concerning the overall prevalence of iron deficiency in the investigated period were tested with a chi-square test. Statistical significance level was set at a two-sided *p*-value < 0.05. Post hoc tests were performed when appropriate. As this is an exploratory data analysis and *p*-values serve only descriptive purposes, no further multiplicity corrections were applied.

## Results

A total of 283 patients were included. The ratio of female to male patients represented in this study was 3:1, with the majority being premenopausal women. 74.2% of the patients underwent one anastomosis gastric bypass (OAGB) and 25.8% of the patients underwent Roux-en-Y gastric bypass (RYGB). Types of operative procedure were comparable between the groups (Table 1). Preoperative patient characteristics are shown in Table 1.

**Table 1:** Type of surgery and patient characteristics

	Men (*n* = 71)		Women premenopausal (*n* = 130)		Women postmenopausal (*n* = 82)		*p*-value*
		*n*		*n*		*n*	
Type of surgery (*n*, %)							
OAGB	56 (78.9%)	71	94 (72.3%)	130	60 (73.2%)	82	
RYGB	15 (21.1%)		36 (27.7%)		22 (26.8%)		
Age (years)	44.3 ± 11.3	71	35.9 ± 8.6	130	54.7 ± 7.0	81	< 0.001^°^
Weight (kg)	147.2 ± 20.3	70	123.1 ± 18.1	130	119.8 ± 15.9	81	< 0.001^+^
BMI (kg/m^2^)	46.5 ± 6.0	70	44.7 ± 5.7	130	45.8 ± 6.7	81	0.06
6 months post surgery	34.4 ± 4.7**	27	30.9 ± 5.3**	40	32.9 ± 4.4**	22	
12 months post surgery	31.0 ± 4.8**	26	28.9 ± 5.2**	46	31.0 ± 6.4**	31	
18 months post surgery	29.4 ± 4.4**	14	28.4 ± 4.9**	39	29.2 ± 5.4**	37	
24 months post surgery	29.7 ± 4.0**	19	27.5 ± 2.6**	27	28.7 ± 5.4**	22	
30 months post surgery	30.2 ± 4.4**	16	26.8 ± 4.4**	19	29.9 ± 4.9**	16	
36 months post surgery	29.8 ± 0.8	4	25.80 ± 3.5**	19	31.6 ± 6.2**	9	
%TWL							
6 months post surgery	28.5 ± 6.5	27	30.1 ± 7.1	40	27.2 ± 7.6	22	
12 months post surgery	33.6 ± 8.0	26	35.3 ± 9.1	46	32.0 ± 7.7	31	
18 months post surgery	38.4 ± 7.4	14	36.7 ± 8.5	39	35.6 ± 7.1	37	
24 months post surgery	35.7 ± 7.2	19	37.4 ± 7.1	27	37.5 ± 9.3	22	
30 months post surgery	35.9 ± 6.3	16	41.5 ± 7.5	19	37.0 ± 7.7	16	
36 months post surgery	35.2 ± 8.6	4	41.9 ± 6.3	19	33.3 ± 10.5	9	

*n* number of patients

*Statistical analysis preoperatively; calculated using Kruskal Wallis test, post hoc test if *p* < 0.05

°Post hoc tests: all pairwise comparisons *p* < 0.001

^+^Post hoc tests: premenopausal women vs. men (*p* < 0.001), postmenopausal women vs. men (*p* < 0.001), premenopausal women vs. postmenopausal women (*p* = 0.376)

**p < 0.05, calculated using Wilcoxon signed rank test, pairwise comparisons between pre- and postoperative BMI

We observed a significant reduction of the BMI at all time points compared to baseline, except for the men 36 months after surgery, which might be due to the small patient number at this time point (Table 1). The biggest weight loss was achieved during the first 6 months for all groups, the weight of the premenopausal women continued to decline over the whole investigation period, whereas the weight of the postmenopausal women and men showed a tendency to rise again after 24 and 18 months, respectively, which can be also seen in the %TWL (Table 1).

**Fig. 2:**
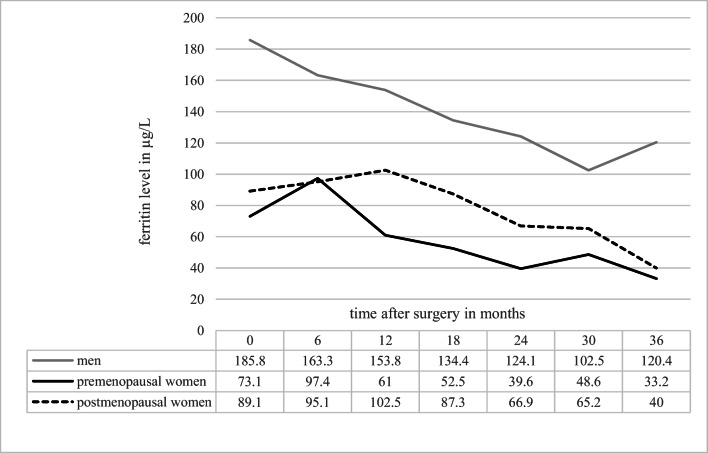
Mean ferritin values in μg/L after surgery

The mean ferritin showed an overall decreasing trend during the investigation period for all three groups (Fig. 2). In accordance with this decline, the prevalence of ferritin values below the reference range showed a drastic increase. Employing group-specific reference ranges based on the laboratory of the General Hospital Vienna, a comparable prevalence of iron deficiency was observed in pre- and postmenopausal women at the investigated periods. The overall prevalence of iron deficiency in the investigated period of 36 months was 36.2% in premenopausal women, 30.5% in postmenopausal women and 11.3% in men. Results of a chi-square test showed a significant difference between the three groups in their prevalence of developing iron deficiency (*p* = 0.001). Men differed significantly from both pre- and postmenopausal women, whereas surprisingly no significant difference could be found between the women before and after menopause (Table 2).

**Table 2:** Prevalence of iron deficiency based on individual ferritin levels below the group-specific reference range

Time after surgery (months)	Men		Women premenopausal		Women postmenopausal	
	Def. (%)	*n*	Def. (%)	*n*	Def. (%)	*n*
0	0	35	1.4	73	9.1	33
6	0	31	9.1	44	17.9	28
12	3.2	31	21.6	51	8.6	35
18	5.3	19	36.6	41	20.5	39
24	13.6	22	32.1	28	39.1	23
30	5.9	17	35.0	20	33.3	18
36	33.3	6	68.4	19	54.5	11
Overall prevalence*	11.3	71	36.2	130	30.5	82

*n* total number of patients

*Chi-square test *p*-value = 0.001

Post-hoc tests: pre- vs. postmenopausal women (*p* = 0.396); premenopausal women vs. men (*p* < 0.001); postmenopausal women vs. men (*p* = 0.004)

No relationship could be observed between the type of surgery and the prevalence of iron deficiency in the investigated period of 36 months. 28.1% of the patients who underwent OAGB had iron deficiency at least at one point during postoperative follow-up versus 28.8% of the patients who underwent RYGB.

Of note, the largest proportion of patients with excess ferritin levels could be observed in premenopausal women at all investigated periods. The prevalence was highest preoperatively (premenopausal women 13.7%, postmenopausal women 3.0%, men 5.7%) and declined continuously in the following months. 85.9% of the men, 96.9% of the premenopausal women and 93.9% of the postmenopausal women had a record of supplementation at least at one visit postoperatively. The mean iron intake overall was 69.08 ± 58.75 mg per day in premenopausal women, 53.60 ± 56.15 mg per day in postmenopausal women and 43.53 ± 39.61 mg per day in men.

## Discussion

Comparing the assessed prevalence of iron deficiency based on the ferritin level with other studies is very difficult due to different cut-off values. But regardless of the cut-offs used, the prevalence of iron deficiency increases in nearly every study, as it does in this investigation [9–11]. Thirty-six months after surgery, 33.3% of the men, 68.4% of the premenopausal and 54.5% of the postmenopausal women showed a ferritin level below the reference value, indicating that the present supplementation regimen is not sufficient to prevent iron deficiency in the patients after bariatric surgery.

There are currently no standardized dietary guidelines for the nutritional management of patients that undergo bariatric surgery [12]. Table 3 summarizes the different recommendations concerning the intake of elemental iron after bariatric surgery.

**Table 3:** Guidelines and recommendations of elemental iron supplementation

Guideline	Prophylaxis of iron deficiency	Treatment of iron deficiency
IFSO-EC/EASO [13]	No exact recommendation	
AACE/TOS/ASMBS/OMA/ASA [14]	45 - 60 mg iron/day	150–200 mg iron up to 2–3 times/day
BOMSS [15]	45–60 mg iron/day Menstruating women: 100 mg/day	
Endocrine Society [16]	--	60 mg iron 2–3 times/day + vitamin C

*IFSO-EC* International Federation for the Surgery of Obesity and Metabolic Disorders – European Chapter, *EASO* European Association for the Study of Obesity, *AACO* American Association of Clinical Endocrinologists, *TOS* The Obesity Society, *ASMBS* American Society for Metabolic & Bariatric Surgery, *OMA* Obesity Medicine Association, *ASA* American Society of Anesthesiologists, *BOMSS* British Obesity and Metabolic Surgery Society

Our current study indicates that the iron substitution of our patients as recommended in electronic health records is not sufficient. Despite the routine recommendation at discharge after surgery to supplement 70 mg of elemental iron, the mean iron intake based on electronic health records was lower during follow-up. The reasons for preferring standard multivitamin and mineral supplements over supplements specialized for patients after bariatric surgeries might be the exclusive availability through online markets and the greater costs of the latter ones compared to standard supplements. Considering all follow-up visits with documented supplementation, premenopausal women did not reach the recommended threshold of at least 45 mg iron supplementation per day in 23.7% of the cases, and 12.4% did not take in any iron at all. Postmenopausal women took in less than 45 mg iron per day in 40.2% and men in 38.5% of the cases. They did not take in any iron at all in 9.8% and 11.5% of the cases, respectively.

**Fig. 3:**
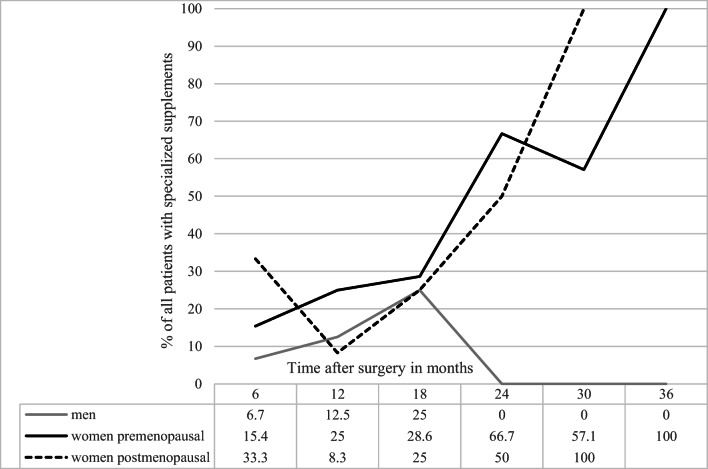
Prevalence of patients taking specialized supplements (70 mg iron) and develop iron deficiency and/or are in need of additional iron supplementation during the analysed period

Previous studies show that iron dosages in standard multivitamin and mineral supplementation (up to 21 mg) are not sufficient to prevent the development of iron deficiency in most patients [6, 17–19]. Our centre recommends supplements specialized for bariatric surgery patients, containing 70 mg of elemental iron. Schijns et al. showed that the mean ferritin serum concentration after bariatric surgery was higher in patients who took specialized supplements with 70 mg iron per day than in those taking standard supplements or no multivitamins. Fewer users of specialized supplements developed iron deficiency compared to non-users. However, in the study, no comparison of men and women was performed. After the first year, a decrease in the mean serum ferritin concentration could be observed in both the users and the non-users, leading to the suggestion that 70 mg iron daily might not be sufficient for at least some patients [20]. This is in line with the data in this study, as there was an increasing prevalence of iron deficiency or the need of an additional iron supplement in female patients who took a supplement containing 70 mg iron a day over time. The men on the other hand showed only a small prevalence for developing iron deficiency when taking specialized supplements (Fig. 3).

The highest prevalence of excess ferritin levels in this study was observed before surgery. Without the state of chronic or acute inflammation, ferritin is the best indicator of iron deficiency [21]. Ferritin binds iron and is stored intracellularly. The little amount that is secreted into the serum correlates strongly with the intracellular concentration of iron [22]. Using ferritin as an indicator of excess ferritin levels, however, has its limitations. Ferritin acts as an acute-phase protein and therefore increases during inflammation [23], and it has been shown that obesity is associated with a low-grade chronic inflammation [24]. The mean CRP value of all groups before surgery was 1.94 mg/dL (reference < 0.5 mg/dL). After surgery, the mean CRP was below the reference value at every time point.

Despite exceeding the reference intakes of the normal population by more than 7 times with the supplementation of at least 70 mg of iron per day [25], no postmenopausal woman and only one man at 6 months post surgery had elevated ferritin values. The premenopausal women exceeded their reference intake by more than 3 times [25] but had a higher prevalence of patients with elevated ferritin levels, although it also remained relatively low (data not shown). It has to be mentioned, however, that the patient numbers were little and even decreased over the investigated period.

Including all follow-up records with documented supplementation, on average, men in this study took in 43.53 mg of iron per day, and pre- and postmenopausal women took in 69.08 mg and 53.60 mg iron per day, respectively. The increasement of the prevalence of patients with iron deficiency suggests that all cohorts should take in a higher amount of iron. Considering the low percentage of excess ferritin levels in both pre- and postmenopausal women, the risk of iron overload even with a higher intake of iron supplements is low.

In this present analysis, different cut-off values for the three groups were used to define iron deficiency. Some authors claim that there is no objective evidence for different reference ranges for men and women as there is no evidence that women have lower requirements of iron [26]. When applying a cut-off value for ferritin of 30 μg/L instead of 15 μg/L to define iron deficiency in premenopausal women, the difference between the cohorts becomes more apparent. Thirty-six months after surgery, the prevalence of iron deficiency of the premenopausal women rises from 68.4 to 78.9%. Other authors however showed that haematological parameters indicating a state of iron deficiency changed at higher serum ferritin levels in postmenopausal women than they did in premenopausal women. Therefore, the authors propose using different cut-off values of ferritin in pre- and postmenopausal women [27].

In general, premenopausal women are at higher risk of developing iron deficiency and subsequently iron deficiency anaemia due to menstrual blood losses [28], with women suffering from heavy menstrual bleeding having an even higher risk [29]. It has been reported that premenopausal women develop iron deficiency more often during postoperative follow-up compared to postmenopausal women and men [30, 31]. This was found to be true in our investigation, too; however, the present study surprisingly indicates a similar prevalence of iron deficiency in pre- and postmenopausal women that differed significantly from the prevalence in men.

In the general population, the mean values of ferritin in women strongly increase after the cessation of menstrual periods. But the accumulation of iron happens gradually with iron-levels of postmenopausal women being still lower than those of men even decades after menopause; therefore, not completely replenished iron stores might be the reason for the higher prevalence of iron deficiency in postmenopausal women compared to men [32, 33]. Figure 2 reveals a comparable relative decrease of the mean ferritin values over time for the three groups. However, men started at a higher level than both pre- and postmenopausal women preoperatively, and 36 months post surgery, the mean ferritin value remained well within the reference range. Although many studies show women to be more iron deficient than men after bariatric surgery [34], the focus in most studies lies mainly on the high prevalence of iron deficiency in premenopausal women. The fact that men and postmenopausal women are often classified as having a “low risk” of developing iron deficiency in the normal population [35] might be the reason for the assumption that these cohorts are not at high risk of developing iron deficiency after gastric bypass surgery as well.

Taken together, our study indicates that due to the apparent diverse need of amounts of iron, an adaption of the dosing regimens not only based on the sex but also on the age should be established. As mentioned above, an adaption for premenopausal women, being at the highest risk of developing iron deficiency, can be found in some guidelines but not in all of them (Table 3). No guideline providing a special dosing regimen for postmenopausal women could be found, even though there seem to be more postmenopausal women iron deficient than men.

A limitation of this study was the retrospective character. The information about the amount of supplements taken was mainly based on self-reports that do not necessarily always have to coincide with objective reality [36]. Despite the attempt to find missing data, the data record remained partially incomplete. Furthermore the number of patients adhering to their aftercare appointments decreased over time, and also the group sizes of the analysed population differ from one another, both limiting factors for the validity.

However, the retrospective study reflects the true clinical situation and makes the results applicable for daily clinical practice.

## Conclusion

Iron deficiency after gastric bypass surgery is very common highlighting that optimized protocols for iron supplementation are needed. Also men and women before and after menopause differ in terms of their iron needs. The high prevalence of iron deficiency in postmenopausal women in this study that is similar to the one of the premenopausal women indicates that there should be put greater focus on this former cohort.

The prevalence of excess ferritin levels is very small at all observed time points and groups and decreases over time, even when getting high amounts of iron; therefore, iron over-supplementation does not appear to be a major problem after gastric bypass surgery. However, further randomized studies on dosing regimens of iron supplementation should be conducted to develop exact recommendations for men and women before and after menopause.
